# Amiodarone-Induced Multi-Systemic Toxicity Involving the Liver, Lungs, Thyroid, and Eyes: A Case Report

**DOI:** 10.3389/fcvm.2022.839441

**Published:** 2022-02-28

**Authors:** Hye-Su You, Jae Hyun Yoon, Sung Bum Cho, Yoo-Duk Choi, Yung Hui Kim, Wonsuk Choi, Ho-Cheol Kang, Sung Kyu Choi

**Affiliations:** ^1^Department of Gastroenterology and Hepatology, Chonnam National University Hospital and Medical School, Gwangju, South Korea; ^2^Department of Gastroenterology and Hepatology, Hwasun Chonnam National University Hospital and Medical School, Hwasun, South Korea; ^3^Department of Pathology, Chonnam National University Hospital and Medical School, Gwangju, South Korea; ^4^Department of Ophthalmology, Chonnam National University Hospital and Medical School, Gwangju, South Korea; ^5^Department of Endocrinology, Hwasun Chonnam National University Hospital and Medical School, Hwasun, South Korea

**Keywords:** amiodarone, toxicity, liver, lung, thyroid

## Abstract

**Objectives:**

Amiodarone is widely used to treat arrhythmia. However, amiodarone is known for its severe toxicity to the liver, lungs, and thyroid. Amiodarone causes liver damage ranging from asymptomatic serum aminotransferase elevation to hepatic failure requiring liver transplantation. Although amiodarone toxicity has been reported, its simultaneous multi-organ toxicity is not well-known. Here, we introduce a novel case of multi-systemic amiodarone toxicity involving the liver, lungs, thyroid, and eyes.

**Case Presentation:**

A 61-year-old woman visited the emergency room due to general weakness, nausea, visual disturbance, heat intolerance, and a non-productive cough. The patient had been using clopidogrel and amiodarone due to underlying atrial fibrillation. The total level of bilirubin was 0.71 mg/dL, aspartate aminotransferase was 358 U/L, alanine aminotransferase was 177 U/L, and prothrombin time was 27.1 s. Computed tomography showed diffuse increased liver intensity and scattered hyperattenuated nodular consolidations in both lungs. Transthoracic needle lung biopsy revealed fibrinoid interstitial inflammation with atypical change of type II pneumocytes and intra-alveolar foamy macrophages. In addition, the thyroid-stimulating hormone level was <0.008 μIU/mL, and free thyroxine was 4.67 ng/dL. The thyroid scan showed diffuse homogenous intake of technetium-99 m pertechnetate in both thyroid lobes. The ophthalmologic exam detected bilateral symmetrical corneal deposits in a vortex pattern. With these findings, we could diagnose amiodarone-induced hepatic, pulmonary, thyroid, and ophthalmologic toxicity. Liver function was restored after cessation of amiodarone, and thyroid function was normalized with methimazole administration. However, due to aggravated lung consolidations, systemic steroid treatment was administered, and improvement was seen 1 week after, at the follow-up exam. As her symptoms improved, she was discharged with a plan of steroid administration for 3 to 6 months.

**Conclusions:**

This case implies the possibility of multi-systemic amiodarone toxicity. Thus, the toxicity of amiodarone to multiple organs must be monitored. Prompt cessation of the drug should be considered upon diagnosis.

## Introduction

Amiodarone is an iodine-containing benzofuran derivative classified as a class III antiarrhythmic drug. It is clinically used for the treatment of tachyarrhythmias, including atrial fibrillation and reentrant tachyarrhythmias (1), which is one of the most prevalent types of arrhythmia (2). However, with the increasing use of amiodarone, there have been reports of side effects to various organs, including the lungs, liver, heart, thyroid gland, eyes, skin, and the nervous system (3).

Overall incidence of adverse effects can range from 30 to 90%, and serious side effects can take place in 10 to 26% of patients (1). Pulmonary toxicity is one of the fatal adverse effects of amiodarone, with mortality estimated between 1 and 33% and incidence of approximately 10% shown in previous studies (4). Patients with amiodarone-induced pulmonary toxicity usually present with dyspnea, non-productive cough, malaise, fever, and pleuritic chest pain (5). The most fatal manifestation of pulmonary toxicity is a rapidly progressing diffuse pneumonitis with acute respiratory distress requiring mechanical ventilation with mortality as high as 50 to 100% (6).

About 24% of patients taking amiodarone showed asymptomatic elevations of serum aminotransferase levels (7). Less than 1% of patients in various studies reported developing significant drug-induced liver injury ranging from symptomatic hepatitis and micronodular liver cirrhosis to hepatic failure requiring liver transplantation (8–14).

In addition, about 14–18% of patients taking amiodarone for a long period showed thyroid dysfunction, including hypothyroidism and thyrotoxicosis (15). However, a third of Korean patients developed thyroid dysfunction, and most cases involved hypothyroidism (16). Patients with amiodarone-induced hypothyroidism usually present with fatigue, cold intolerance, and dry skin, similar to symptoms of classic hypothyroidism (17).

The most typical symptom of amiodarone-induced ocular toxicity is corneal microdeposits, 98% of which are found after 2 months of treatment (18). Asymptomatic corneal changes are observed in 50–60% of patients, and visual disturbance is rarely reported (19).

Although much is known about amiodarone toxicity, cases of simultaneous toxicity in various organs have rarely been reported. Here, we present a novel case of multi-systemic amiodarone toxicity, involving the liver, lungs, thyroid, and eyes.

## Case Report

A 61-year-old woman visited the emergency room due to general weakness, nausea, visual disturbance, heat intolerance, and a non-productive cough with dyspnea, which had persisted between 3 and 6 months. The patient had underlying ischemic heart disease and paroxysmal atrial fibrillation, for which she was being treated with clopidogrel, pitavastatin, valsartan, rivaroxaban, nicorandil, diltiazem, furosemide, and amiodarone. More specifically, amiodarone was prescribed for 34 months at an initial dose of 200 mg/day for 11 months, and then due to intermittent chest discomfort with palpitation, her cardiologist increased the maintenance dose to 400 mg/day for the next 23 months. The patient was not taking any other medications, including herbal agents, and did not have a history of alcohol consumption.

**Figure 1:**
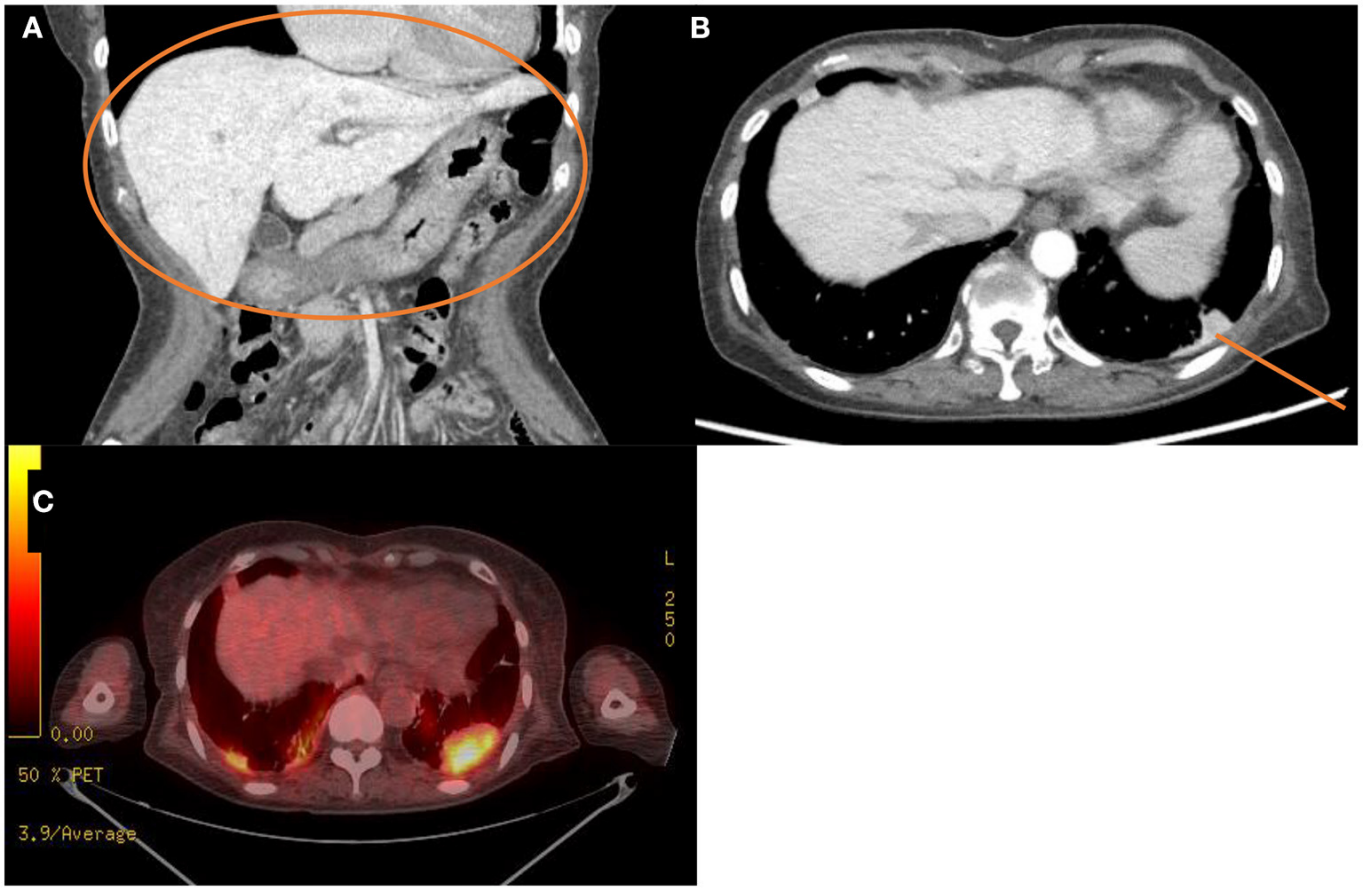
Radiologic findings of the abdominal cavity and the chest. **(A,B)** Initial computed tomography scan of the abdominal cavity and the chest. This scan shows diffuse increased liver intensity [**(A)**, orange circle] and scattered hyperattenuation nodular consolidations in the subpleural areas of both lungs [**(B)**, orange line]. **(C)** Fluorodeoxyglucose positron emission tomography–computed tomography scan of the whole body showing about 4.8 cm hypermetabolic lesion in the left lung field (maximal standardized uptake, 7.3).

**Figure 2:**
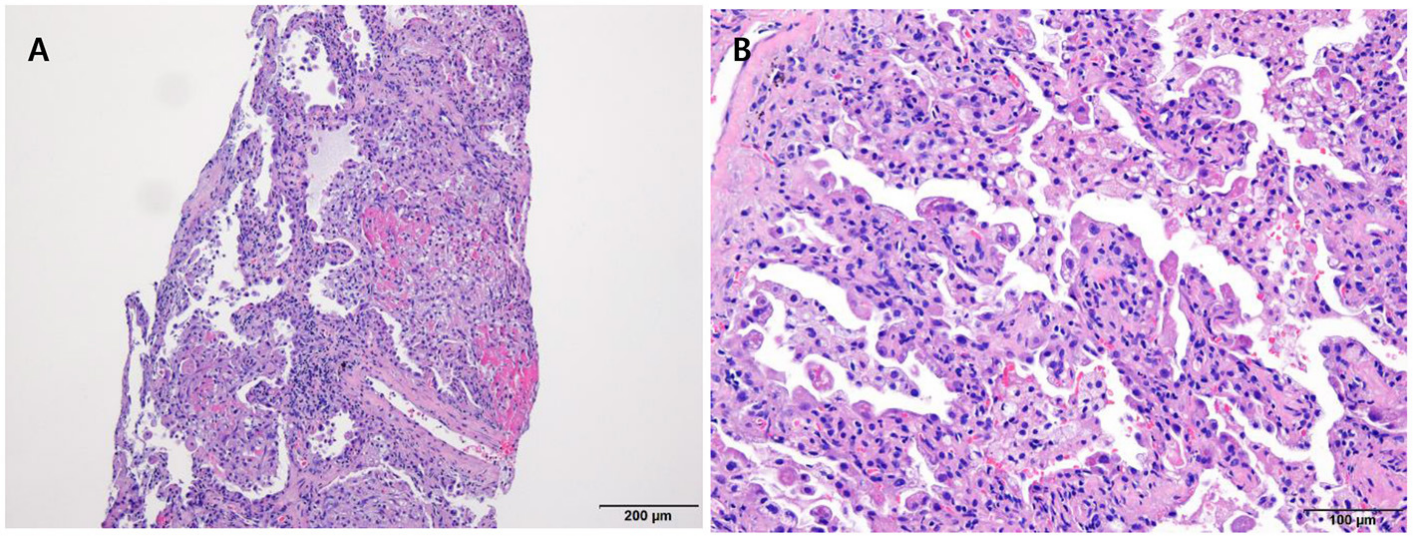
Pathologic examination of the lung via trans-thoracic needle biopsy showing amiodarone-induced lung toxicity. **(A)** Chronic interstitial pneumonitis and interstitial fibrosis and focal fibrinoid necrosis. **(B)** Accumulation of foamy macrophages within alveolar spaces.

The laboratory workup showed that the level of the total bilirubin was 0.71 mg/dL (reference range, 0.22–1.3), the aspartate aminotransferase level was 358 U/L (reference range, 10–37), the alanine aminotransferase level was 177 U/L (reference range, 10–37), and the prothrombin time was 1.90 International Normalized Ratio (INR) (reference range, 0.8–1.2). Enhanced computed tomography (CT) showed diffusely increased liver intensity and scattered hyper attenuated nodular consolidations in the subpleural areas of both lungs (Figures 1A,B). To evaluate the possibility of malignancy, positron emission tomography–CT (PET–CT) was performed, and multiple hypermetabolic lesions (maximal standardized uptake values 7.3, Figure 1C) were examined. To confirm the diagnosis of the lung lesions, transthoracic needle biopsy was performed, and the pathologic examination showed fibrinoid interstitial inflammation with atypical change of type II pneumocytes and intra-alveolar foamy macrophages (Figure 2). Using both the CT and biopsy findings, we could diagnose the amiodarone-induced hepatic and pulmonary toxicity.

**Figure 3:**
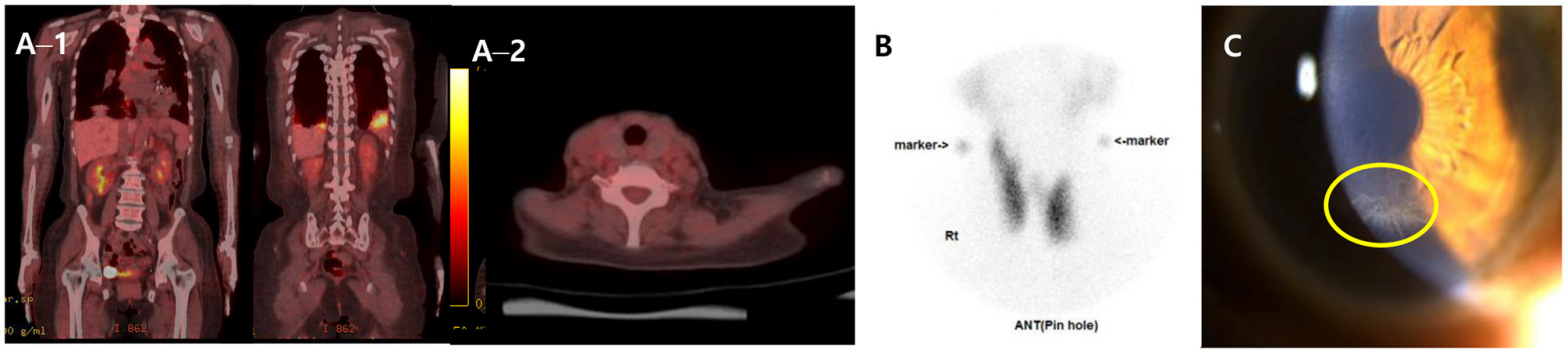
FDG PET–CT, Thyroid scan and ophthalmologic exam. **(A)** FDG PET–CT scans of the whole body (A−1) and the thyroid (A−2) showing multiple hypermetabolic lesion in both lungs and no metabolic lesion in other organ including the thyroid. **(B)** Thyroid scan (technetium-99 m pertechnetate scintigraphy) presenting diffuse homogenous intake of technetium-99 m pertechnetate in both thyroid lobes. **(C)** Slit-lamp biomicroscopy showing numerous dense gray granular lines forming vortex patterns radiating from a median point at the junction of the middle and lower thirds of the cornea. FDG, fluorodeoxyglucose; PET–CT, positron emission tomography–computed tomography.

Further, to assess the possibility of amiodarone-induced thyroid dysfunction, we examined thyroid function tests. The thyroid-stimulating hormone level was less than 0.008 uIU/mL (reference range, 0.55–4.78), free thyroxine was 4.67 ng/dL (reference range, 0.89–1.76), triiodothyronine was 1.27 ng/mL (reference range, 0.6–1.81), thyroid binding inhibitor immunoglobulin (TBII) was 18.3 IU/L (reference range, 0–1.5), anti-microsome antibody was 139.0 U/mL (reference range, 0–60), and anti-thyroglobulin antibody was 125 U/L (reference range, 0–60). The PET–CT scan of the thyroid showed no metabolic lesion (Figure 3A). The thyroid scan (technetium-99 m pertechnetate scintigraphy) showed diffuse homogenous intake of technetium-99 m pertechnetate in both thyroid lobes (Figure 3B). Anti-microsomal antibody and anti-thyroglobulin antibody may be detected in Graves' disease (anti-microsomal antibody: 69.2%, anti-thyroglobulin antibody: 23–30%) (20); furthermore, with the results of TBII and the thyroid scan results, we diagnosed our patient as having type 1 amiodarone-induced thyrotoxicosis, a form of iodine-induced hyperthyroidism caused by excessive, uncontrolled biosynthesis of thyroid hormone by autonomously functioning thyroid tissue in response to iodine load, which typically develops in underlying latent Graves' disease (21). After consultation with the endocrinology department, methimazole (20 mg for 17 days followed by 5 mg/d) was used as treatment to manage excessive thyroid hormone synthesis.

The patient persistently complained of blurred vision, and the ophthalmologic exam detected bilateral symmetrical corneal deposits in a vortex pattern (Figure 3C), which was compatible with amiodarone-induced vortex keratopathy.

**Figure 4:**
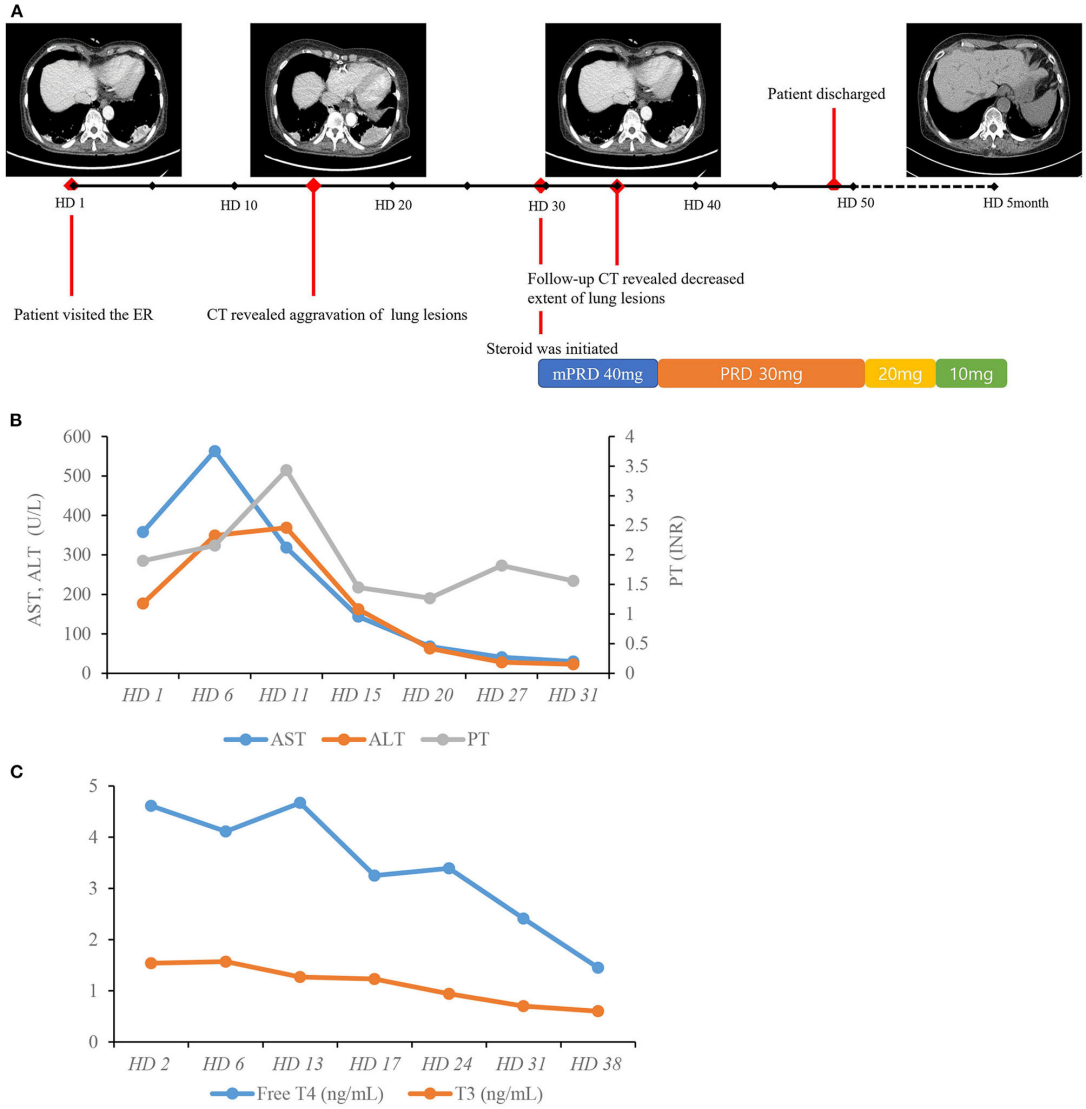
Hospital course of the patient. **(A)** Timeline of chest CT scans and resolution of lung lesions with systemic steroid therapy. **(B)** Course of AST, ALT, and PT. **(C)** Course of free T4 and T3. AST, aspartate aminotransferase; ALT, alanine aminotransferase; HD, hospital day; INR, international normalized ratio; PT, prothrombin time; TSH, thyroid-stimulating hormone; T4, thyroxine; T3, triiodothyronine.

As amiodarone toxicity was considered as the possible cause of disease, amiodarone was discontinued from the first day of admission. As there was neither extensive lung involvement nor hypoxemia, steroid treatment was not immediately initiated. However, serial follow-up chest CT scans showed aggravation of previous lung lesions. After exclusion of lung malignancy via transthoracic needle biopsy, we decided on treatment with a systemic steroid regimen (methylprednisolone 40 mg) for pulmonary toxicity from day 29 of hospitalization. After 1 week of steroid medication, a follow-up CT showed an overall decreased extent of high attenuated subpleural mass-like consolidations and small nodules in both upper lung fields and the right middle lobe, suggesting an improved state of R/O amiodarone-induced pulmonary toxicity. After 4 months of steroid medication, a follow-up CT showed resolution of previous lung consolidations (Figure 4A). The laboratory examination showed that both aspartate aminotransferase and alanine aminotransferase levels were within the normal range after cessation of amiodarone for 1 month without any treatment (Figure 4B). Free thyroxine and thyroid-stimulating hormone were within the normal range after 6 weeks of methimazole medication (Figure 4C). The patient recovered from her general weakness, cough, and hand tremor 1 month after amiodarone cessation and was discharged after most of the symptoms were resolved.

## Discussion

To the best of our knowledge, this is the first report demonstrating multi-systemic toxicity, involving four vital organs, induced by amiodarone. Amiodarone is a widely prescribed antiarrhythmic drug used to treat tachyarrhythmias, including atrial fibrillation and reentrant tachyarrhythmias of the accessory pathways. Amiodarone principally acts through multiple mechanisms, such as depressing the sinus and atrioventricular nodes and prolonging repolarization and refractoriness in the myocardium.

Amiodarone is a lipophilic drug, which accumulates mainly in adipose tissue and organs with high blood perfusion, such as the liver, lungs, and skin. Its long-term use leads to numerous adverse reactions, including photosensitivity, hypothyroidism, hyperthyroidism, hepatic dysfunction, bone marrow suppression, corneal microdeposits, and neuromotor defects (3). Due to its long biological half-life ranging up to 100 days, the drug activity may last more than 3 to 4 months after discontinuation (1).

Hepatotoxicity is a relatively uncommon adverse reaction to amiodarone. Amiodarone induces histological findings similar to alcohol-induced steatohepatitis, so differential diagnosis from alcoholic liver disease should be considered. Pathologic findings include macro- and microvesicular steatosis, Mallory bodies, polymorphonuclear leukocyte infiltration, ballooning degeneration of hepatocytes and phospholipidosis (22). The possible reason for hepatotoxicity is amiodarone, as its principal metabolite, desethylamidoarone induces production of reactive oxygen species and leads to hepatic triglyceride accumulation and microvesicular steatosis in hepatocytes (23). Pulmonary toxicity, an uncommon but serious adverse effect of amiodarone, reveals various patterns of lung involvement, including chronic interstitial pneumonia, bronchiolitis with or without organizing pneumonia, acute respiratory distress syndrome, diffuse alveolar hemorrhage, pleural effusion, and pulmonary nodules or masses. Especially, pulmonary mass and pulmonary nodules can mimic lung malignancy (24). The mechanism underlying the development of nodular pulmonary disease may be the inflammatory response to the accumulation of phospholipids in alveolar cells induced by the drug (25). Corticosteroid therapy should be considered in patients showing extensive lung involvement in imaging or those that develop hypoxemia, with a starting dose of 40 to 60 mg/day of prednisolone (26, 27). Due to the structural similarity between amiodarone and thyroid hormones, amiodarone causes thyroid dysfunction due to excessive iodine overload or due to its direct cytotoxicity to the thyroid gland (28). Amiodarone can induce both hypothyroidism and hyperthyroidism. The incidence of amiodarone-induced thyrotoxicosis is 2 to 10%, and it more commonly develops in areas of the world where iodine deficiency is common (29). The type of thyroid dysfunction caused by amiodarone treatment may depend on the presence of underlying thyroid disease or dietary iodine content (30). Moreover, amiodarone leads to drug-induced lipidosis, which leads to typical side-effects in the eyes, such as vortex keratopathy (31). Although the most common findings are corneal and lens opacities, optic neuropathy has also been reported (32). There have been reports of amiodarone toxicity to one or two vital organs. Cho et al. (33) reported a case of a 65-year-old man with amiodarone-induced hepatitis and hypothyroidism. Turk et al. (34) also reported a case of a 54-year-old female with amiodarone toxicity to the skin, thyroid, and eyes.

In this study, we observed multi-systemic amiodarone toxicity involving the liver, lungs, thyroid, and eyes. The main mechanism of amiodarone-induced toxicity is known as direct cytotoxicity and immunologic reaction. Patients who have received a daily dose of 400 mg or more for more than 2 months or a lower dose for more than 2 years are considered at high risk (26). Our patient can be considered as high-risk, as she was on 400 mg/day amiodarone for nearly 2 years. The long duration and high dosage of amiodarone were combined with treatment that may interact pharmacologically with amiodarone metabolism. First of all, she had been taking a statin concurrently with amiodarone for 3 years. Many studies have reported an interaction between statins and amiodarone as the potential cause of hepatotoxicity due to inhibition of the mitochondrial enzyme CYP3A4, which metabolizes statins, by amiodarone (35–38). Additionally, the patient in this study took diltiazem, a recognized CYP3A4 inhibitor that may have jeopardized amiodarone metabolism. Furthermore, our patient showed significant improvement of lung lesions with the use of systemic steroid therapy, suggesting the usefulness of corticosteroids in extensive lung involvement, as reported in previous studies (26, 27).

Our study has some limitations. First, although our report indicates multi-systemic amiodarone toxicity, the number of cases examined was very small. Second, the precise mechanism of amiodarone toxicity to multiple vital organs is not clearly verified. Lastly, the drug interactions associated with amiodarone metabolism, which may be the cause of amiodarone toxicity in our patient, had not been thoroughly examined. Further studies are warranted to elucidate the mechanism of multi-organ toxicity and classify high-risk patients to prevent amiodarone-induced toxicity.

In conclusion, this case indicates that multi-systemic amiodarone organ toxicity is a cause of concern in high-risk patients. Amiodarone toxicity should be considered in patients receiving amiodarone with any new symptoms, because this may involve various organs. A multisystem approach to amiodarone toxicity is important, because adverse events may occur in various organs simultaneously. Guidelines for monitoring adverse events in patients taking amiodarone for a long time must be established. Once diagnosis is determined, amiodarone must be discontinued promptly, and the use of systemic steroids needs to be assessed.

## Data Availability Statement

The original contributions presented in the study are included in the article/supplementary material, further inquiries can be directed to the corresponding author/s.

## Author Contributions

H-SY and JY conceived and designed the study, reviewed the literature, and contributed to manuscript drafting. SBC and SKC contributed to manuscript drafting. JY reviewed the case and edited the manuscript. Y-DC reviewed the pathologic findings. YK reviewed the ophthalmologic findings. WC and H-CK contributed to interpretation of data and reviewed of the case regarding amiodarone-induced thyrotoxicosis. All authors approved the final version to be submitted and approved the publication of the manuscript.

## Funding

This study was supported by a grant from the Chonnam National University Hospital Biomedical Research Institute (BCRI21069) and National Research Foundation of Korea (2021R1F1A1061719).

## Conflict of Interest

The authors declare that the research was conducted in the absence of any commercial or financial relationships that could be construed as a potential conflict of interest.

## Publisher's Note

All claims expressed in this article are solely those of the authors and do not necessarily represent those of their affiliated organizations, or those of the publisher, the editors and the reviewers. Any product that may be evaluated in this article, or claim that may be made by its manufacturer, is not guaranteed or endorsed by the publisher.
